# Development and Validation of 58K SNP-Array and High-Density Linkage Map in Nile Tilapia (*O. niloticus*)

**DOI:** 10.3389/fgene.2018.00472

**Published:** 2018-10-15

**Authors:** Rajesh Joshi, Mariann Árnyasi, Sigbjørn Lien, Hans Magnus Gjøen, Alejandro Tola Alvarez, Matthew Kent

**Affiliations:** ^1^Department of Animal and Aquacultural Sciences, Faculty of Biosciences, Norwegian University of Life Sciences, Ås, Norway; ^2^Genomar Genetics AS, Trondheim, Norway

**Keywords:** Nile tilapia, linkage map, SNP array, genomics, sex determination, amh, anti-Müllerian hormone

## Abstract

Despite being the second most important aquaculture species in the world accounting for 7.4% of global production in 2015, tilapia aquaculture has lacked genomic tools like SNP-arrays and high-density linkage maps to improve selection accuracy and accelerate genetic progress. In this paper, we describe the development of a genotyping array containing more than 58,000 SNPs for Nile tilapia (*Oreochromis niloticus*). SNPs were identified from whole genome resequencing of 32 individuals from the commercial population of the Genomar strain, and were selected for the SNP-array based on polymorphic information content and physical distribution across the genome using the Orenil1.1 genome assembly as reference sequence. SNP-performance was evaluated by genotyping 4991 individuals, including 689 offspring belonging to 41 full-sib families, which revealed high-quality genotype data for 43,588 SNPs. A preliminary genetic linkage map was constructed using Lepmap2 which in turn was integrated with information from the O_niloticus_UMD1 genome assembly to produce an integrated physical and genetic linkage map comprising 40,186 SNPs distributed across 22 linkage groups (LGs). Around one-third of the LGs showed a different recombination rate between sexes, with the female being greater than the male map by a factor of 1.2 (1632.9 to 1359.6 cM, respectively), with most LGs displaying a sigmoid recombination profile. Finally, the sex-determining locus was mapped to position 40.53 cM on LG23, in the vicinity of the anti-Müllerian hormone (amh) gene. These new resources has the potential to greatly influence and improve the genetic gain when applying genomic selection and surpass the difficulties of efficient selection for invasively measured traits in Nile tilapia.

## Introduction

Nile tilapia (*Oreochromis niloticus*) is an important fresh-water aquaculture species farmed in more than 100 countries including many developing countries in which the species is an essential source of dietary protein (ADB, 2005). Thanks to its fast growth, short generational interval (5 months), relatively small size, adaptability to different environments, and easy to handle, it is also used as a model species for research in fish endocrinology (Seale et al., 2002), physiology (Wright and Land, 1998; Vilela et al., 2003), and evolutionary and developmental biology (Fujimura and Okada, 2007). Nile tilapia production is supported by more than 20 breeding programs based mainly in South East Asia and some in Africa and America (Neira, 2010). Most of the commercial and farmed Nile tilapia strains are derived from the genetically improved farmed tilapia (GIFT) base strain established in the early 1990s (Eknath et al., 1993). Among these, the Genomar Supreme Tilapia (GST^®^) strain which has undergone more than 25 generations of selection.

So far Nile tilapia breeding programs have relied on traditional breeding approaches based on easily measurable phenotypes such as weight and length, and have just recently started to implement modern genome-based strategies, such as marker-assisted and genomic selection (personal communication). Compared to livestock species, aquaculture has been slower to adopt genome-based selection tools largely due to a lack of genomic resources such as reference genomes, SNP arrays, and linkage maps. But in species like rainbow trout, salmon, and common carp where genomic selection is being practiced, priority of utilizing genomic information is on selection for disease and parasitic resistance. For example, resistance against Bacterial Coldwater Disease (BCWD) (Vallejo et al., 2015a,b, 2017b), infectious pancreatic necrosis (IPN) (Yoshida et al., 2018) and *Piscirickettsia salmonis* (Yoshida et al., 2017a) in rainbow trout; *Piscirickettsia salmonis* (Bangera et al., 2017) and resistance against sea lice (Ødegård et al., 2014; Tsai et al., 2016; Correa et al., 2017a) in Atlantic salmon; *Piscirickettsia salmonis* in coho salmon (Barría et al., 2018); and juvenile growth rate in common carp (Tsai et al., 2015b; Palaiokostas et al., 2018). Similarly, lots of GWAS studies have been conducted in these species, primarily for disease resistance (Correa et al., 2015; Liu et al., 2015; Palti et al., 2015; Vallejo et al., 2017a; Barría et al., 2018), resistance against sea lice (Davidson and Yáñez, 2016; Correa et al., 2017b), sexual maturity (Ayllon et al., 2015; Gutierrez et al., 2015) and some carcass quality traits (Sodeland et al., 2013; Tsai et al., 2015a; Gonzalez-Pena et al., 2016; Yoshida et al., 2017b).

The first genome assembly for *O. niloticus* (released in 2011; Orenil1.0, and updated to Orenil1.1 at the end of 2012 (NCBI, 2018)) was based on short-read sequencing. A newer assembly (O_niloticus_UMD1) was generated using a combination of novel long-reads (generated using Pacific Biosciences Technology) and publicly available Illumina short reads (Conte et al., 2017). Four linkage maps of varying resolution were constructed using markers found with Restriction-site Associated DNA (RAD) sequencing (Palaiokostas et al., 2013), microsatellites and/or AFLP markers (Kocher et al., 1998; Lee et al., 2005; Guyon et al., 2012). The RAD based strategies usually generate a SNP resource of medium density and are highly efficient in species where a reference genome is not available (Robledo et al., 2017). In comparison, a SNP-array offers the advantages of increased genotype accuracy of much higher numbers of markers as well as control over the physical distribution of these across the genome (Robledo et al., 2017). In this paper, we report the development of a 58K SNP-array (Onil50) and construction of a high density linkage map in the commercial strain of Nile tilapia, Genomar Supreme Tilapia (GST^®^), which is the continuation of the widespread GIFT-strain.

## Materials and Methods

### SNP-Array (Onil50) Development

#### Origin of Sequenced Fish

**FIGURE 1:**
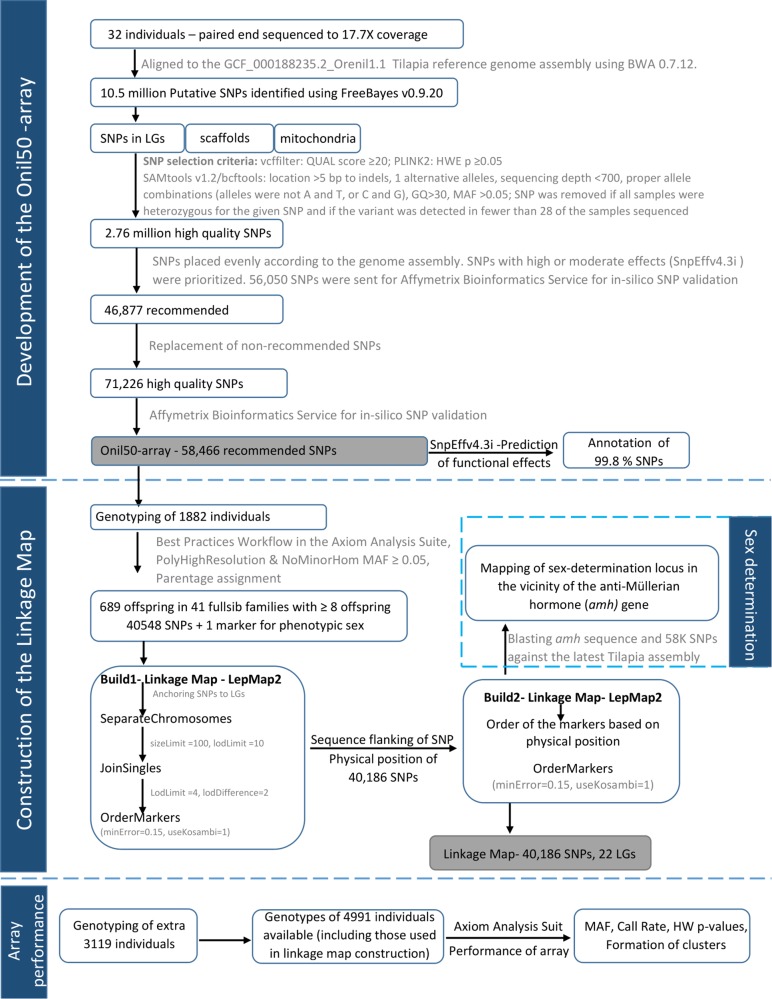
The graphical summary of the pipeline for array design, validation, and linkage map construction.

The GST^®^ strain of Nile tilapia used in this study originates from the original GIFT population (Eknath et al., 1993). This strain was selected for growth from generation 1 to 14, growth and filet yield from generation 15 to 19, and growth, yield, and robustness from generation 20. Thirty-two individuals (13 males and 19 females) from this population were selected for whole genome sequencing. Twenty of them are from generation 23, selected at random from the breeding nucleus and the rest 12 are from a commercial line formed from generation 20 and selected for growth (**Supplementary Figure 6**). The graphical summary of the methodology is given in **Figure 1**.

#### Whole Genome Sequencing and SNP-Detection

Genomic DNA from these 32 individuals was extracted from fin-clips (preserved in Ethanol) using Qiagen DNeasy columns (Qiagen, Germany). DNA quality was assessed by agarose gel electrophoresis and quantified using a Qubit fluorometer (Thermo Fisher Scientific, United States). After normalization, sequencing libraries were prepared and barcoded using TruSeq sample preparation kit and sequenced (2 × 125) across 10 lanes on an Illumina HiSeq 2500 (Illumina, United States) by a commercial provider. At the time this work was carried out, Orenil1.1 Tilapia represented the highest quality reference genome available (NCBI Assembly *Oreochromis niloticus*: GCF_000188235.2_Orenil1.1_genomic), and reads were aligned to it using BWA-MEM algorithm in BWA 0.7.12 (Li, 2013) with default parameters. Putative SNPs were identified using FreeBayes v0.9.20 (Garrison and Marth, 2012) with parameters genotype-qualities and experimental-gls. Using vcffilter, SNPs with a QUAL score value (phred) of ≤20 were removed.

#### SNP-Filtering

The initial set of putative SNPs was divided into three groups including SNPs located on scaffolds assigned to linkage groups (LGs) of the assembly, SNPs on unassigned scaffolds, and SNPs detected within the mitochondrial genome. SAMtools v1.2/bcftools (Li et al., 2009) was then used to filter out variants according to the following criteria: a SNP was removed if; (i) located within 5 bp to an indel, (ii) had more than one alternative allele, (iii) the sequencing depth exceeded 700 reads, (iv) its alleles were A and T, or C and G (these require twice as many ‘probes’ on Affymetrix SNP arrays as other SNP allele combinations), (v) if sample genotype quality (GQ) was <30, (vi) minor allele frequency (MAF) <0.05, (vii) all samples were heterozygous for the given SNP, (viii) the variant was detected in fewer than 28 of the samples sequenced. Finally, Hardy–Weinberg Equilibrium (HWE) exact test was calculated using PLINK2 (Chang et al., 2015) and SNPs that showed departure from HWE (*P* < 0.05) were removed.

#### SNP-Selection

After filtering, 2.76 million putative high-quality SNPs remained. Based on their relationship to genes and physical distribution, a subset of these was identified for inclusion on the array. SNPEff v 4v1l (Cingolani et al., 2012) was used to identify SNPs with high and moderate effects (including for example non-synonymous variants). The SNPs with high effects are assumed to have disruptive impact in the protein codification, probably causing protein truncation, loss of function or triggering non-sense mediated decay, e.g., stop gained, frameshift variant, etc.; whereas the SNPs with moderate effects are assumed to be non-disruptive, but they might change protein effectiveness, e.g., missense variant, inframe deletion, etc. From the list of almost 38,000 variants with high and moderate effects, approximately 10,000 were chosen avoiding SNPs within 10 kb of another. A python script was used to fill in gaps and produce a relatively even distribution of SNPs selected at ≈12 kb intervals across the 22 LGs, and ≈33 kb across unmapped scaffolds >50 kb in length. The script was designed to fill a distribution gap with a variant falling within a small size selection window with highest MAF being the main criteria. SNPs from the mitochondrial genome were selected manually. The selected subset of SNPs (*n* = 56,050) were submitted to *in silico* validation for Affymetrix Bioinformatic Service. The Affymetrix *in silico* probe set design and evaluation pipeline predicts the performance of SNPs and calculates a conversion probability value (*p*-convert value: representing the probability of a given SNP converting to a reliable SNP assay on the Axiom array system) using various criteria including: binding energy, GC content, and the expected degree of non-specific hybridization to multiple genomic regions. Based on the *p*-convert values, they classify the SNPs into different categories: recommended, neutral, not-recommended, etc. 46,877 SNPs under the categories recommended and/or neutral from probe scoring recommendations were retained and 24,349 extra SNPs were chosen from the regions were the SNPs were discarded. A total of 71,226 SNPs (46,877 + 24,349) were sent back to Affymetrix for *in silico* SNP validation. Finally, the best 58,466 SNPs were chosen to tile on the array based on their probe scoring recommendation (at least one of the strand were recommended, or got neutral category). Upon its release, SNP positions were redefined based on the O_niloticus_UMD1 assembly (Conte et al., 2017) using NCBI’s Genome Remapping Service.

### Construction of Genetic Map

#### Genotyping

Genomic DNA was isolated from ethanol-preserved fin clips collected from 1882 Nile tilapia samples using Qiagen 96 DNeasy Blood & Tissue Kits according to manufacturer’s instructions (Qiagen, Germany) for map construction. These samples were from different generations of GST^®^ strain within the same breeding population. After quantification and quality checking of DNA, samples were genotyped on the Onil50 array at Center for Integrative Genetics (CIGENE) in Norway.

The unfiltered dataset contained 58,466 SNPs, which were analyzed using the Best Practices Workflow with default settings (sample Dish QC ≥ 0.82, QC call rate ≥97; SNP call-rate cutoff ≥97) in the Axiom Analysis Suite software. Ten samples were excluded from analyzed dataset because of the low call rate. Then, the SNPs classified as PolyHighResolution or NoMinorHom [most informative categories from Best Practices Workflow in Axiom Analysis Suite software (Thermo Fisher Scientific Inc, 2018)] were selected, leaving us with 43,014 SNPs.

#### Family Structure

The 1872 genotyped individuals could be divided into two groups based on the generations of the breeding population. Group 1 (*n* = 1124) comprised individuals collected following the branching of the 20th generation, and were factorially crossed against each other after two generations. The experimental design for Group 1 is described in Joshi et al. (2018) and was primarily intended to partition the non-additive genetic effects in this population. Fish from Group 2 (*n* = 748) were obtained from the 24th and 25th generations of GST^®^ (**Supplementary Figure 6**).

Parentage assignment was done using an exclusion method which eliminates animals from a list of potential parents when there are opposing homozygotes between parents and offspring (Hayes, 2011). We used all the 43,014 SNPs and permitted a maximum of 100 conflicts between parents and offspring, representing approximately 0.24% of all genotypes. A total of 689 offspring was divided among 41 full-sib families containing ≥8 offspring (mean offspring per family, μ = 16.81). Group 1 (468 offspring with 19 parents) had 34 full-sib families (μ = 13.77 ± 5.5) and Group 2 (221 offspring with 14 parents) had 7 full-sib families (μ = 31.57 ± 7.23). The structure of Groups 1 and 2 is shown in **Supplementary Tables 1**, **2**.

#### Linkage Map Construction

The SNPs displaying a MAF ≤0.05 (2,466 SNPs) were further filtered for linkage map construction. All the retained SNPs (*n* = 40,548) had SNP call rate >0.97, so this criteria was not used for filtration. Phenotypic sex was known for a subset of families (221 offspring + 33 parents) and was coded as 12 for males and 11 for females and included in the genotype file (*n* = 40,549) before running Lepmap2 (Rastas et al., 2013) for linkage map construction. Lepmap2 uses information from full-sibs and their parents to assign SNP markers to LGs, and applies standard hidden Markov model (HMM) to compute the likelihood of the marker order within each LGs. First, the SNPs were used to construct the preliminary linkage map (Build 1), which was used to anchor, order, and orient the scaffolds in the O_niloticus_UMD1 assembly and upgrading this assembly to O_niloticus_UMD_NMBU (Conte et al., 2018). Eventually, the final physical integrated genetic linkage map (Build 2) was constructed from the order of the markers based on the physical position of the SNPs in O_niloticus_UMD_NMBU assembly.

#### Build 1: To Anchor SNPs to Different LGs

SeparateChromosomes (a module in Lepmap2) was run testing lodLimits from 1 to 50 and a sizeLimit = 100; a lodLimit of 10 resulted in 22 LGs, also with the lowest number of markers not assigned to any LG. JoinSingles was run to assign single markers to LG groups and tested with lodLimits from 1 to 15 and lodDifference = 2; a lodLimit of 4 was selected as this joined the highest number of single markers. OrderMarkers was used to order the markers within each LG. Each LG was ordered separately and replicated 5 times with commands: numThreads = 10, polishWindow = 30, filterWindow = 10, useKosambi = 1, minError = 0.15, and the order with highest likelihood was selected as the best order. For sex averaged map OrderMarkers was run similarly by adding sexAverage = 1.

#### Build 2: Integrated Linkage Map Based on the Order of the SNPs in the New Assembly

Sequence containing each SNP was used to find the physical position of the SNPs in the O_niloticus_UMD_NMBU assembly. Physical position information was used to adjust the order of the SNPs within respective LGs and Lepmap2 was rerun to produce the final linkage map.

### Array and SNP-Performance

To get a more comprehensive overview about the array and SNP performance, 3119 additional Nile tilapia samples were genotyped using Onil50 array. The raw dataset of the 1872 samples which were used for linkage mapping was combined with the dataset of the 3119 samples and were analyzed together using the Best Practices Workflow with default parameters in Axiom Analysis Suite software (Thermo Fisher Scientific Inc, 2018). Four quality parameters were assessed on those samples filtered through Dish QC (DQC ≥ 0.82), QC call rate (QC CR ≥ 97) and plate QC (Percent of passing samples ≥50 and average call rate for passing samples ≥50) criteria: MAF, SNP call rates, Hardy Weinberg (HW) *p*-values, and clustering. SNPs could be divided into six different types on the basis of formation of clusters (i) “PolyHighResolution” – formation of three clusters with good resolution; (ii) “NoMinorHom” – formation of two clusters with no samples of one homozygous genotype; (iii) “MonoHighResolution” – a single cluster of a homozygous genotype; (iv) “OTV,” off-target variants – three good clusters, with a single additional off-target cluster caused by variants in the SNP flanking region; (v) “CallRateBelowThreshold” – the SNP call rate was below the threshold (0.970), but other cluster properties were above the threshold; and (vi) “Other” – the SNPs were not grouped into any of the previous categories.

SnpEffv4.3i (Cingolani et al., 2012) was used to predict functional effects of the 58,340 SNPs which were remapped to O_niloticus_UMD1 assembly.

## Results

### SNP Selection and Array Development

The sequencing of 32 Nile tilapia generated 4.22 × 10^9^ reads representing an average of 17.7× coverage per individual (stdv = 4.2, min = 9.4, and max = 27.7). After alignment, on average 98% of reads were mapped to the Orenil1.1 assembly yielding 12.78 million variants of which 10.5 million were putative SNPs. Rest 2.2 million were insertions, deletions, multi-nucleotide polymorphisms, etc. After performing multiple steps of filtering described in the section “Materials and Methods,” a subset of 2.76 million SNPs was retained and a final set of 58,466 SNPs were selected for assay design and printed on the Onil50 array.

Around 99.8% of the SNPs from the array were successfully re-mapped to the new O_niloticus_UMD1 assembly (**Table 1**). Remapping revealed an increase in the number of SNPs mapping to LGs and a corresponding decrease in the number of SNPs on unmapped scaffolds. The average variant density per LG on the Orenil1.1 assembly is 12.5 ± 0.35 kb (12.1–13.7 kb). However, since the O_niloticus_UMD1 assembly includes an additional 87 Mb assigned to LGs the average variant density increased to 15.5 ± 4.06 kb (13.3–32.3 kb) (**Table 1**). Additional information about inter-SNP distance and standard deviation can be found in **Supplementary Table 4**. Physical size of LG03 increased by 2.4 times in the new assembly, thereby increasing the number of SNPs assigned to this LG by 2.3 times.

**Table 1:** Sequence similarity based assignment of SNPs contained on the Onil50-array to Orenil1.1 and O_niloticus_UMD1 genome assemblies.

Orenil1.1 assembly				O_niloticus_UMD1 assembly			
LG	Length (bp)	Variants	Variants rate (bp/variant)	LG	Length (bp)	Variants	Variants rate (bp/variant)
LG01	31194787	2571	12133	LG01	38372991	2830	13559
LG02	25048291	2043	12261	LG02	35256741	2395	14721
LG03	19325363	1415	13658	LG03	68550753	2105	32565
LG04	28679955	2288	12535	LG04	38038224	2427	15673
LG05	37389089	2927	12774	LG05	34628617	2549	13585
LG06	36725243	2891	12703	LG06	44571662	2932	15202
LG07	51042256	4128	12365	LG07	62059223	4682	13255
LG08–24	29447820	2314	12726	LG08	30802437	2307	13352
LG09	20956653	1732	12100	LG09	27519051	1909	14415
LG10	17092887	1414	12088	LG10	32426571	1878	17267
LG11	33447472	2653	12607	LG11	36466354	2662	13699
LG12	34679706	2753	12597	LG12	41232431	2833	14554
LG13	32787261	2647	12387	LG13	32337344	2275	14214
LG14	34191023	2700	12663	LG14	39264731	2679	14656
LG15	26684556	2180	12241	LG15	36154882	2255	16033
LG16–21	34890008	2777	12564	LG16	43860769	2848	15401
LG17	31749960	2609	12169	LG17	40919683	2873	14243
LG18	26198306	2075	12626	LG18	37007722	2307	16041
LG19	27159252	2223	12217	LG19	31245232	2301	13579
LG20	31470686	2491	12634	LG20	36767035	2635	13953
LG22	26410405	2083	12679	LG22	37011614	2272	16290
LG23	20779993	1603	12963	LG23	44097196	2225	19819
Total	657350972	52517	12517	Total	868591263	56216	15451
Unmapped scaffold (*n* = 557)	246010115	5939	41422	Unmapped scaffolds (*n* = 284)		2151	
Mitochondrial genome	16627	10	1662	Mitochondrial genome	16627	10	
Total number of SNPs on the array		58466		Remapped SNPs in total		58340	
				SNPs failed to remap		126	

### Performance and Validation of the SNPs in the Array

**FIGURE 2:**
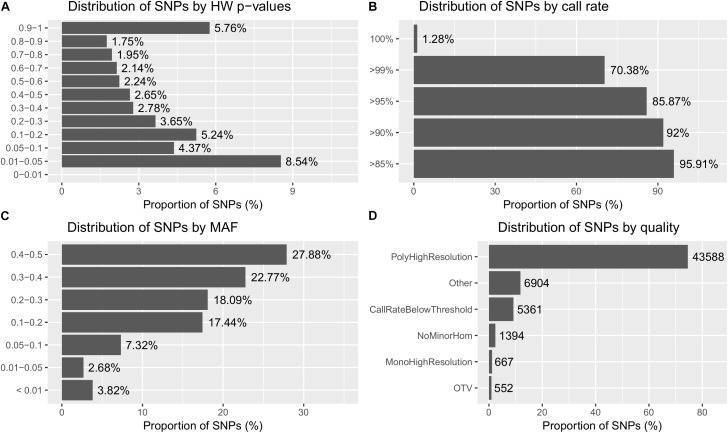
Summary of the SNP metrics based on 58,466 SNPs on 4,947 GST^®^ samples after being subjected to sample call rate in Best Practices Workflow of Axiom Analysis Suite software (see methodology). **(A)** Distribution of SNPs based on HW *p*-values obtained from the exact HWE test. **(B)** Distribution of SNPs based on SNP call rate. **(C)** Distribution of SNPs based on minor allele frequency (MAF). **(D)** Distribution of SNPs based on types of cluster formation (quality). The different types of clusters are based on the quality of the SNPs and has been described in the methodology.

A total of 4947 samples out of 4991 passed the Dish QC threshold. A total of 4858 samples (97.3%) were left after being subjected to sample call rate. Based on the cluster profile, over 74% of the 58,466 SNPs were classified as PolyHighResolution. More detailed information about the sample and SNP statistics are shown in **Figure 2**.

Prediction of functional effects of the 58,340 remapped SNPs from the Onil50 array resulted, in most cases, in multiple annotations per variant. The effects with the highest putative impact are included for summary in **Table 2**. The majority of the SNPs are intronic (36.92%) or intergenic (20.80%) variants, while about 15% of are non-synonymous mutations. Since the SNP selection process specifically targeted variants with a potential functional effect, these variants are expected to have direct effect on traits of interest.

**Table 2:** Summary of annotation for SNPs in the Onil50-array.

SNP categories	Count	Percent
Total number of SNPs in the array	58,446	
Annotation possible	58,340	99.82
Annotation results		
Nonsense-mediated decay (NMD)	19	0.03
Loss of function (LOF)	114	0.20
Intergenic region	12,156	20.80
Intragenic variant	126	0.22
Intron variant	21,581	36.92
Non-synonymous variant		
Missense variant	8,765	15.00
Missense variant & splice region variant	263	0.45
Stop gained	16	0.03
Stop lost	13	0.02
Synonymous variant	1,142	1.95
Non-coding transcript exon variant	27	0.05
Splice acceptor variant & intron variant	9	0.02
Splice donor variant & intron variant	13	0.02
Splice region variant	8	0.01
Splice region variant & intron variant	163	0.28
Splice region variant & non-coding transcript exon variant	7	0.01
Splice region variant & synonymous variant	28	0.05
Upstream gene variant	9,231	15.79
3 prime UTR variant	1,533	2.62
5 prime UTR premature start codon gain variant	21	0.04
5 prime UTR variant	459	0.79
Downstream gene variant	2,646	4.53

### Linkage Map

A total of 40,549 SNPs were retained following quality filtering, and 99.78% of these (*n* = 40,467) were ordered within the 22 LGs corresponding to the karyotype of Nile tilapia (**Supplementary Figure 1**). Since, Build 1 linkage map is an intermediate step for the extension of the O_niloticus_UMD1 to the O_niloticus_UMD_NMBU genome assembly (Conte et al., 2018), which is not the aim of this paper, we give only a brief summary of the results. The genetic and physical maps were generally in good agreement with a correlation of ≥0.96 between the reference genome position and the genetic map position of the SNPs (**Supplementary Figure 1**). This high correlation with the physical map demonstrates that the genetic map is of high quality and is highly accurate.

**FIGURE 3:**
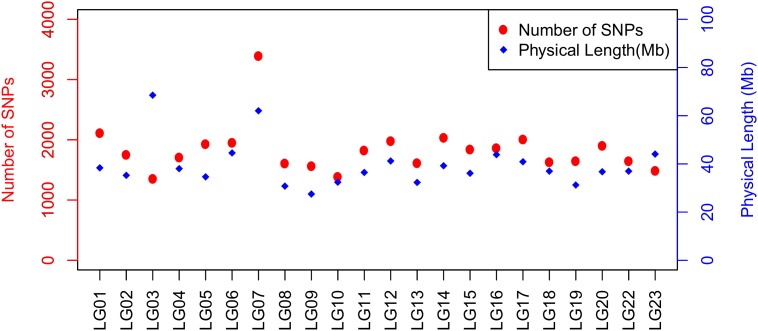
Plot illustrating the number of SNPs and physical length of LG based on O_niloticus_UMD1 Assembly and Build 2 linkage map.

**FIGURE 4:**
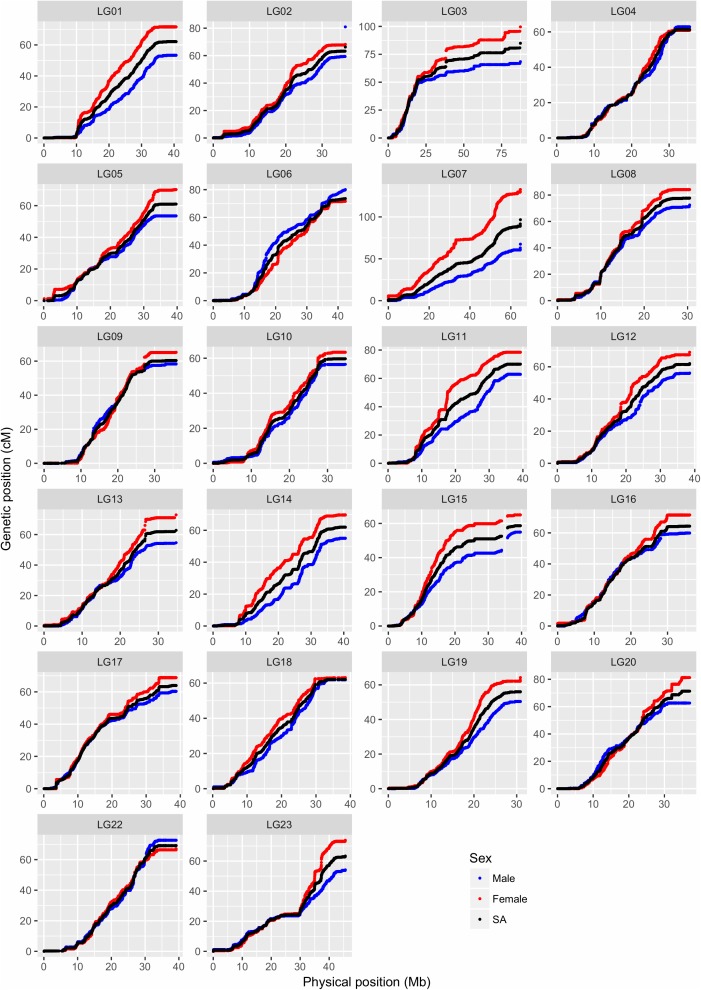
Comparison of map positions between genetic and physical maps for different LGs in Build 2. The *y*-axis gives the linkage map positions, and the *x*-axis gives the physical positions. Linkage groups (LGs) and the physical positions are based on O_niloticus_UMD_NMBU Assembly. The maps are color-coded: red for female specific, blue for male specific, and black for sex-averaged linkage maps. Please note that the 22 LGs in the Nile tilapia have been named from LG01 to LG23 (no LG21).

A total of 40,186 SNPs mapped to 22 LGs in Build 2 linkage map. The consensus (sex-averaged) map adds up to 1469.69 cM, with individual LG lengths ranging from 56.04 cM (LG19) to 96.68 cM (LG07) (**Table 3**). The average genetic distance across the LGs was 66.8 cM. The number of markers per LG varied from 1349 to 3391, with an average of 1827 markers per LG (**Table 3**). As a consequence of the SNP selection, which sought to position a SNP every 12 kb, the number of markers was mostly proportional to the size of the LG (**Figure 3**). A notable exception is LG03 where the inclusion of previously unassigned scaffolds has trippled the physical size without a corresponding tripling of SNP numbers. The SNP density (SNPs/Mb) varied across the genome from 19.68 to 56.83 (see also **Figure 4** and **Supplementary Figures 2–4**).

**Table 3:** Marker numbers, length, density, and correlations for male, female, and sex-averaged Build 2 linkage map.

LG	No. of SNPs	Physical length^1^	Length (cM)			(F:M length)	Marker density per Mb	Marker density per cM			Correlation^2^		
			F	M	SA			F	M	SA	F	M	SA
LG01	2112	38.37	71.64	53.35	62.11	1.34	55.04	29.48	39.59	34	0.98	0.98	0.99
LG02	1749	35.26	68.22	80.97	66.27	0.84	49.60	25.64	21.6	26.39	0.98	0.99	0.99
LG03	1349	68.55	99.59	68.38	84.91	1.46	19.68	13.55	19.73	15.89	0.91	0.84	0.88
LG04	1707	38.04	60.9	62.85	61.39	0.97	44.87	28.03	27.16	27.81	0.99	0.98	0.99
LG05	1925	34.63	70.2	53.59	61.02	1.31	55.59	27.42	35.92	31.55	0.99	0.99	0.99
LG06	1948	44.57	71.66	80.09	73.6	0.89	43.71	27.18	24.32	26.47	0.99	0.98	0.99
LG07	3391	62.06	132.69	67.45	96.68	1.97	54.64	25.56	50.27	35.07	0.99	0.99	0.99
LG08	1607	30.80	84.18	72.48	77.72	1.16	52.18	19.09	22.17	20.68	0.98	0.98	0.99
LG09	1564	27.52	65.26	58.34	60.39	1.12	56.83	23.97	26.81	25.9	0.97	0.97	0.98
LG10	1387	32.43	63.42	56.51	59.69	1.12	42.77	21.87	24.54	23.24	0.98	0.97	0.98
LG11	1821	36.47	78.49	62.87	70.07	1.25	49.93	23.2	28.96	25.99	0.97	0.99	0.99
LG12	1979	41.23	69.03	56.09	61.99	1.23	48.00	28.67	35.28	31.92	0.98	0.99	0.99
LG13	1614	32.34	72.9	54.64	62.79	1.33	49.91	22.14	29.54	25.7	0.99	0.99	0.99
LG14	2030	39.26	69.67	55.02	61.99	1.27	51.71	29.14	36.9	32.75	0.99	0.98	0.99
LG15	1836	36.15	65	54.95	58.68	1.18	50.79	28.25	33.41	31.29	0.95	0.97	0.96
LG16	1862	43.86	71.61	59.94	64.36	1.19	42.45	26	31.06	28.93	0.99	0.98	0.99
LG17	2005	40.92	68.88	60.36	63.97	1.14	49.00	29.11	33.22	31.34	0.98	0.97	0.98
LG18	1628	37.01	63.14	61.85	62.1	1.02	43.99	25.78	26.32	26.22	0.99	0.99	1
LG19	1646	31.25	64.21	50.37	56.04	1.27	52.67	25.63	32.68	29.37	0.98	0.99	0.98
LG20	1899	36.77	81.26	62.64	71.31	1.3	51.65	23.37	30.32	26.63	0.99	0.99	0.99
LG22	1643	37.01	67.09	72.69	69.25	0.92	44.39	24.49	22.6	23.73	0.98	0.98	0.98
LG23	1484	44.10	73.86	54.17	63.36	1.36	33.65	20.09	27.4	23.42	0.96	0.98	0.97
Total	40186	868.59	1632.9	1359.6	1469.69								
Average	1827	39.48	74.22	61.80	66.80	1.20	47.41	24.61	29.56	27.34	0.98	0.98	0.98

*^*1*^The physical length (Mb) is retrieved from O_niloticus_UMD1 Assembly. ^*2*^The correlation between the genetic distance of SNPs (cM) on the linkage map and the physical distance (bp) according to the reference genome assembly. F, M, and SA represents female, male, and sex-averaged maps, respectively*.

In this study, paternal and maternal informative markers were used to construct specific male and female maps (**Table 3**). Around one-third of the LGs showed a different recombination rate between sexes (**Supplementary Figure 5**), with male and female map lengths differing by a factor of 1.2 (1359.6 and 1632.9 cM, respectively). Generally, female maps were found to be larger in all LGs with the exception of LG02, LG06, and LG22. Sigmoidal pattern of recombination, with no recombination at both ends of the LGs, was seen in almost all LGs (**Figure 4**).

## Discussion

### High-Density Linkage Map for Nile Tilapia

Existing linkage maps for Nile tilapia contain relatively few markers unevenly distributed across LGs (**Supplementary Table 3**). As a consequence, regions in the genome have poor SNP coverage. By stringently selecting SNPs with an even physical distribution in the genome the linkage map presented includes 10 times more SNPs and fewer gaps, compared to the most recent map (Palaiokostas et al., 2013).

Ferreira et al. (2010) categorized the karyotypes of *O. niloticus* into 3 meta-submetacentric and 19 subtelo-acrocentric chromosomes. The steepness of the curve in **Figure 4** shows the recombination level, with flat lines representing little or no recombination, which may suggest the possible location of the centromeres. There are some discontinuities present in LG03 and LG15, suggesting that our linkage map lacks the SNPs in those regions. These gaps might be due to missing sequence in the assembly or due to the assembly errors. Wide recombination deserts (areas with no recombination) are seen in the initial and/or end regions of most of the LGs, generally up to 5 Mb and sometimes up to 10 Mb (e.g., LG09 and LG10), indicating the presence of subtelo-acrocentric chromosomes. Because of these recombination deserts, most of the LGs, irrespective of the sexes, showed sigmoidal pattern, which is unusual when compared to other fish species. In channel catfish (Li et al., 2014), salmon (Tsai et al., 2015b), Asian seabass (Wang et al., 2015) and stickleback (Roesti et al., 2013) the recombination rates were generally elevated toward the end of the LGs. The possible explanation might be that the GST^®^ strain used in this study is derived from the GIFT strain, formed from crossing among four wild and four cultured Asian strains (Eknath et al., 1993). When the individuals from two different strains are crossed together, the offspring is heterozygous and causes difficulty in recombination producing stretch with low recombination, which might have resulted to the unique recombination pattern. Low recombination at the end of LGs were also observed in the hybrid crosses of Lake Malawi cichlids (Conte et al., 2018).

Nile tilapia was shown to have a sex-specific pattern of recombination with the female map generally being larger than the male map (Lee et al., 2004). The genetic basis for the differences in the recombination rate between sexes has still not been found, but Li et al. (2014) has listed three major hypotheses. First, the selection perspective hypothesis (Lenormand and Dutheil, 2005; Gruhn et al., 2013), proposes that the selection pressure is higher in male compared to female gametes during the haploid life stage and this male-specific selection leads to decrease in the male recombination rate to maintain the beneficial haplotypes. Second, the compensation hypothesis (Coop and Przeworski, 2007), proposes that the recombination rate is higher in females compared to males to compensate for the less stringent checkpoint for the non-recombinant (achiasmatic) chromosomes. Third, the recombination pathway hypothesis (Gruhn et al., 2013), suggests that the chromatin differences established prior to the onset of the recombination pathway causes the differences in the recombination between the two sexes.

LG23 showed a unique recombination pattern, a flat line of around 5 Mb, in the center of the LG, for which there also is a sex difference in recombination rate. In *O. niloticus*, major XY sex determining regions have earlier been mapped to LG1 (Palaiokostas et al., 2013) and LG23 (Karayücel et al., 2004; Shirak et al., 2006; Eshel et al., 2011, 2012). Further, tandem duplication of the variants of the gene anti-Müllerian hormone (*amh)* in LG23 has been identified as the male sex determinant in Nile tilapia (Li et al., 2015). These variants of *amh* gene have been mapped to around 35.4 Mb region of Nile tilapia genome (discussed below in section “Sex Locus Mapped in the Vicinity of *amh* Gene”), which is the same region where the unique recombination pattern is seen, suggesting limited recombination around the sex-determining genes in *O. niloticus*. Further, LG23 was formed by the fusion of two LGs during the evolution of cichlids (Liu et al., 2013), which might be another reason for this unique recombination pattern.

The fusion of the LGs during the evolutionary process also has an effect on the size of the LGs, as it is believed that the ancestors of cichlids had 24 chromosome pairs, which eventually became 22 pairs (Majumdar and McAndrew, 1986; Ferreira et al., 2010). Our genetic map shows that LG07 is the largest, which has been shown to be formed by the fusion of two LGs during lineage evolution (Poletto et al., 2010; Liu et al., 2013; Conte et al., 2017).

### Array Content and Performance

SNP performance was validated by genotyping around 5000 individuals from different generations of the GST^®^ strain of Nile tilapia. Around 75% of the SNPs on the array perform well generating three highly differentiated allelotype clusters (i.e., PolyHighResolution). Around 9% of the SNPs were found to depart from HWE (*p* < 0.01), but it has to be noted that the population genotyped for the validation is the commercial strain that has undergone up to 25 generations of selection. Hence, these departures might be important as they could represent regions under selection, domestication and the outcome of assortative mating (Gutierrez et al., 2016; Adenyo et al., 2017). Whereas the extreme departures might suggest lethal recessive mutations and/or recent mutations or copy number variants (Lee et al., 2008; Graffelman et al., 2017).

For future revisions, the array could be improved by increasing the SNP density in highly recombinant regions of specific LGs like including LG03 and LG23. The use of genetic distance rather than the physical distance to select the SNPs is probably the best option for equidistant SNP distribution across the genome.

### Sex Locus Mapped in the Vicinity of *amh* Gene

Sex determination is one of the important aspect in commercial tilapia production, as males are found to grow faster than females and unisex production is a main method to avoid propagation in production ponds or cages. Sex determination in fish is more complicated than mammals as it tends to be dependent on both genetic and environmental factors (Ezaz et al., 2006; Baroiller et al., 2009). Besides hermaphrodite species, two main sex determination system exist: XY and ZW, and they are both present in fish species. It has also been seen that phylogenetically closely related fish species, even in same genus, have different sex determination systems. For example, Blue tilapia, *Oreochromis aureus*, has the ZW sex determination system (Campos-Ramos et al., 2001), where males are homogametic (ZZ) and females are heterogametic (ZW), so the ovum determines the sex of the offspring. On the other hand, Nile tilapia (*O. niloticus*) and Mozambique tilapia (*O. mossambicus*) have the XY system of sex determination, where the males are heterogametic (XY) and females are homogametic (XX), so the sperm determines the sex of the offspring (Mair et al., 1991; Campos-Ramos et al., 2003).

In our study, the sex locus for Nile tilapia was coded using the XY system and mapped to LG23 (**Table 4**) as reported previously in several studies (Karayücel et al., 2004; Shirak et al., 2006; Eshel et al., 2011, 2012). SNP AX-164998274 (SNP probe: AGGTGTGTGGTCTTTCTTTGGAAGTCTGCAGAGTG[C/T]TTCAATAACACAGGTATGGTTTCTCGTTGTGATTC) mapped to the same genetic position as the sex locus. The most likely position of sex locus (pos. 34.5 Mb/40.53 cM on LG23) maps close to the anti-Müllerian hormone (*amh*) gene, previously characterized as sex determining gene in Nile tilapia (Li et al., 2015).

**Table 4:** Mapping of sex-determination locus in the vicinity of the anti-Müllerian hormone (*amh)* gene.

SNPs/gene	LG	Position (bp)	Male (cM)	Female (cM)	Average (cM)
AX-165032341	LG23	34305951	35.03	44.83	39.97
AX-164990538	LG23	34306186	35.03	44.83	39.97
AX-165017655	LG23	34319855	35.03	44.83	39.97
AX-165032969	LG23	34336514	35.03	44.83	39.97
AX-165012489	LG23	34351488	35.03	44.83	39.97
AX-164995826	LG23	34367182	35.24	44.83	40.00
AX-165001648	LG23	34380102	35.45	44.83	40.09
AX-165030187	LG23	34380282	35.45	44.85	40.12
AX-164992183	LG23	34398468	35.45	44.88	40.13
AX-165006758	LG23	34424845	35.45	44.95	40.15
AX-164986178	LG23	34437472	35.45	45.02	40.20
AX-165024637	LG23	34451454	35.45	45.61	40.53
AX-165013086	LG23	34465412	35.45	45.61	40.53
AX-164998274^∗^	LG23	34496900	35.45	45.61	40.53
amh_delta-y	LG23	34491516-34499598			
amhy	LG23	34491516-34503495			
amh	LG23	34491516-34509687			
AX-164990628	LG23	34510978	35.45	45.61	40.53
AX-165031999	LG23	34511701	35.46	45.61	40.54
AX-165013176	LG23	34525091	35.46	45.61	40.54
AX-165010851	LG23	34576386	35.46	45.61	40.54
AX-164993854	LG23	34585587	35.46	45.61	40.54
AX-164989444	LG23	34598712	35.46	45.61	40.54

*SNP AX-164998274 (marked as ^∗^) mapped to the same genetic position as the Phenotypic sex of the individuals in the Build 1 linkage map.*

### Implications in Tilapia Industry

Tilapia is a commercially important aquaculture species, with more than 3.9 million tons of fish and filets being traded in 2015 (FAO, 2017) and more than 20 breeding programs (Neira, 2010). The present SNP array and linkage map has the potential to greatly improve the genetic gain for this economic important species, and help surpass the difficulties of efficient selection for the invasively measured traits, the traits which cannot be measured directly on the candidate broodstock fish, but are only measured on the sibs of the candidates, e.g., disease resistance, filet yield, etc. These tools may also be useful to bridge the genotype-phenotype gap in Nile tilapia, which has been pursued for a long time (Gjøen, 2004).

A major capability of these resources will be to find economic important QTLs or chromosome regions affecting economically important traits like disease resistance, filet traits or feed efficiency. In order to fine map these QTLs, it is essential to have a high-resolution linkage map. The dense linkage map can also be integrated with physical maps to position and orient scaffolds along LGs, thereby producing genome assemblies of higher quality.

Another important implication will be to facilitate the shift from traditional breeding strategies to genomic selection in Nile tilapia. In the future, breeding goals in Nile tilapia will include many invasively measured traits. Genomic selection will significantly help us to overcome these challenges, increasing the profitability and the genetic gain (Meuwissen et al., 2001; Nielsen et al., 2009; Sonesson and Meuwissen, 2009; Vela-Avitúa et al., 2015; Hosoya et al., 2017; Houston, 2017). Finally, this will also help to discern more accurately the additive from the non-additive genetic effects, thereby increasing the selection accuracy and the possibility to utilize non-additive genetic effects (Varona et al., 2018).

Another obvious use of the SNP-array will be in the parentage assignments. The drawback of the conventional breeding designs in Nile tilapia using PIT tags is the confounding of the full-sib family effects (due to communal rearing of full-sibs) and maternal environmental effects (due to mouth brooding), making it difficult to detangle the various variance components accurately (Joshi et al., 2018), which ultimately decreases the accuracy of the selection.

## Conclusion

We present the first SNP-array, the Onil50-array, containing ca 58,000 SNPs for Nile tilapia, which was validated in close to 5000 individuals. Further, we constructed a high density integrated genetic and physical linkage map, with LGs showing sex-differentiated sigmoidal recombination patterns. These new resources has the potential to greatly influence and improve the genetic gain when applying genomic selection and surpass the difficulties of efficient selection for invasively measured traits in Nile tilapia.

## Data Availability

The assemblies used in this study can be found in NCBI using the following accessions: Orenil1.1 = GCF_000188235.2, O_niloticus_UMD1 = MKQE00000000, and O_niloticus_UMD_NMBU = MKQE02000000. The whole genome sequence data used for SNP detection has been deposited in the European Nucleotide Archive and can be found in EMBL-EBI_website using accession PRJEB28330. Linkage map generated from this study can be found in the Figshare: https://figshare.com/s/8427b97cf6e623173232.

## Ethics Statement

Sampling of DNA was done in accordance with the commercial practice and norms by Genomar Genetics AS.

## Author Contributions

HG, AA, and MK conceived and designed the study. AA coordinated biological sampling. MK and MÁ were responsible for array design and MÁ performed lab work and initial analysis of results. RJ constructed the linkage map, while SL integrated the genetic and physical maps. RJ and MÁ prepared the draft manuscript which was reviewed and edited by HG, MK, AA, and SL. All authors read and approved the manuscript.

## Conflict of Interest Statement

Genomar Genetics AS employs one of the authors, AA. The remaining authors declare that the research was conducted in the absence of any commercial or financial relationships that could be construed as a potential conflict of interest.

## References

[B1] ADB (2005). *An Impact Evaluation of the Development of Genetically Improved Farmed Tilapia and their Dissemination in Selected Countries.* Mandaluyong: Asian Development Bank.

[B2] Adenyo C. Ogden R. Kayang B. Onuma M. Nakajima N. Inoue-Murayama M. (2017). Genome-wide DNA markers to support genetic management for domestication and commercial production in a large rodent, the Ghanaian grasscutter (*Thryonomys swinderianus*). *Anim. Genet.* 48 113–115. 10.1111/age.12478 27436241

[B3] Ayllon F. Kjærner-Semb E. Furmanek T. Wennevik V. Solberg M. F. Dahle G. (2015). The vgll3 locus controls age at maturity in wild and domesticated Atlantic salmon (*Salmo salar* L.) males. *PLoS Genet.* 11:e1005628. 10.1371/journal.pgen.1005628 26551894 PMC4638356

[B4] Bangera R. Correa K. Lhorente J. P. Figueroa R. Yáñez J. M. (2017). Genomic predictions can accelerate selection for resistance against *Piscirickettsia salmonis* in Atlantic salmon (*Salmo salar*). *BMC Genomics* 18:121. 10.1186/s12864-017-3487-y 28143402 PMC5282740

[B5] Baroiller J.-F. D’Cotta H. Bezault E. Wessels S. Hoerstgen-Schwark G. (2009). Tilapia sex determination: where temperature and genetics meet. *Comp. Biochem. Physiol. Part A Mol. Integr. Physiol.* 153 30–38. 10.1016/j.cbpa.2008.11.018 19101647

[B6] Barría A. Christensen K. A. Yoshida G. M. Correa K. Jedlicki A. Lhorente J. P. (2018). Genomic predictions and genome-wide association study of resistance against *Piscirickettsia salmonis* in coho salmon (*Oncorhynchus kisutch*) using ddRAD sequencing. *G3* 8 1183–1194. 10.1534/g3.118.200053 29440129 PMC5873909

[B7] Campos-Ramos R. Harvey S. C. Masabanda J. S. Carrasco L. A. Griffin D. K. McAndrew B. J. (2001). Identification of putative sex chromosomes in the blue tilapia, *Oreochromis aureus*, through synaptonemal complex and FISH analysis. *Genetica* 111 143–153. 10.1023/A:1013707818534 11841163

[B8] Campos-Ramos R. Harvey S. C. McAndrew B. J. Penman D. J. (2003). An investigation of sex determination in the Mozambique tilapia, *Oreochromis mossambicus*, using synaptonemal complex analysis, FISH, sex reversal and gynogenesis. *Aquaculture* 221 125–140. 10.1016/S0044-8486(03)00072-3

[B9] Chang C. C. Chow C. C. Tellier L. C. A. M. Vattikuti S. Purcell S. M. Lee J. J. (2015). Second-generation PLINK: rising to the challenge of larger and richer datasets. *Gigascience* 4:7. 10.1186/s13742-015-0047-8 25722852 PMC4342193

[B10] Cingolani P. Platts A. Wang L. L. Coon M. Nguyen T. Wang L. (2012). A program for annotating and predicting the effects of single nucleotide polymorphisms, SnpEff: SNPs in the genome of *Drosophila melanogaster* strain w1118; iso-2; iso-3. *Fly* 6 80–92. 10.4161/fly.19695 22728672 PMC3679285

[B11] Conte M. A. Gammerdinger W. J. Bartie K. L. Penman D. J. Kocher T. D. (2017). A high quality assembly of the Nile Tilapia (*Oreochromis niloticus*) genome reveals the structure of two sex determination regions. *BMC Genomics* 18:341. 10.1186/s12864-017-3723-5 28464822 PMC5414186

[B12] Conte M. A. Joshi R. Moore E. C. Nandamuri S. P. Gammerdinger W. J. Clark F. E. (2018). Chromosome-scale assemblies reveal the structural evolution of African cichlid genomes. *bioRxiv* [Preprint]. 10.1101/383992 PMC6447674 30942871

[B13] Coop G. Przeworski M. (2007). An evolutionary view of human recombination. *Nat. Rev. Genet.* 8 23–34. 10.1038/nrg1947 17146469

[B14] Correa K. Bangera R. Figueroa R. Lhorente J. P. Yáñez J. M. (2017a). The use of genomic information increases the accuracy of breeding value predictions for sea louse (*Caligus rogercresseyi*) resistance in Atlantic salmon (*Salmo salar*). *Genet. Sel. Evol.* 49:15. 10.1186/s12711-017-0291-8 28143593 PMC5282780

[B15] Correa K. Lhorente J. P. Bassini L. López M. E. Di Genova A. Maass A. (2017b). Genome wide association study for resistance to *Caligus rogercresseyi* in Atlantic salmon (*Salmo salar* L.) using a 50K SNP genotyping array. *Aquaculture* 472 61–65. 10.1016/j.aquaculture.2016.04.008

[B16] Correa K. Lhorente J. P. López M. E. Bassini L. Naswa S. Deeb N. (2015). Genome-wide association analysis reveals loci associated with resistance against *Piscirickettsia salmonis* in two Atlantic salmon (*Salmo salar* L.) chromosomes. *BMC Genomics* 16:854. 10.1186/s12864-015-2038-7 26499328 PMC4619534

[B17] Davidson W. S. Yáñez J. M. (2016). Genome wide association study for resistance to *Caligus rogercresseyi* in Atlantic salmon (*Salmo salar* L.) using a 50K SNP genotyping array. *Aquaculture* 472 61–65. 10.1016/j.aquaculture.2016.04.008

[B18] Eknath A. E. Tayamen M. M. Palada-de Vera M. S. Danting J. C. Reyes R. A. Dionisio E. E. (1993). Genetic improvement of farmed tilapias: the growth performance of eight strains of *Oreochromis niloticus* tested in different farm environments. *Aquaculture* 111 171–188. 10.1016/B978-0-444-81527-9.50021-X

[B19] Eshel O. Shirak A. Weller J. I. Hulata G. Ron M. (2012). Linkage and physical mapping of sex region on LG23 of Nile tilapia (*Oreochromis niloticus*). *G3* 2 35–42. 10.1534/g3.111.001545 22384380 PMC3276181

[B20] Eshel O. Shirak A. Weller J. I. Slossman T. Hulata G. Cnaani A. (2011). Fine-mapping of a locus on linkage group 23 for sex determination in Nile tilapia (*Oreochromis niloticus*). *Anim. Genet.* 42 222–224. 10.1111/j.1365-2052.2010.02128.x 24725231

[B21] Ezaz T. Stiglec R. Veyrunes F. Graves J. A. M. (2006). Relationships between vertebrate ZW and XY sex chromosome systems. *Curr. Biol.* 16 R736–R743. 10.1016/j.cub.2006.08.021 16950100

[B22] FAO (2017). *FAO Yearbook. Fishery and Aquaculture Statistics. 2015.* Rome: FAO.

[B23] Ferreira I. A. Poletto A. B. Kocher T. D. Mota-Velasco J. C. Penman D. J. Martins C. (2010). Chromosome evolution in African cichlid fish: contributions from the physical mapping of repeated DNAs. *Cytogenet. Genome Res.* 129 314–322. 10.1159/000315895 20606399 PMC3202915

[B24] Fujimura K. Okada N. (2007). Development of the embryo, larva and early juvenile of Nile tilapia *Oreochromis niloticus* (Pisces: Cichlidae). Developmental staging system. *Dev. Growth Differ.* 49 301–324. 10.1111/j.1440-169X.2007.00926.x 17501907

[B25] Garrison E. Marth G. (2012). Haplotype-based variant detection from short-read sequencing. *arXiv* [Preprint]. https://arxiv.org/abs/1207.3907

[B26] Gjøen H. M. (2004). “A new era: the merging of quantitative and molecular genetics—Prospects for tilapia breeding programs,” in *Proceedings of the 6th International Symposium on Tilapia in Aquaculture*, eds Bolivar R. B. Mair G. C. Fitzsimmons K. (Manila: Bureau of Fisheries and Aquatic Resources).

[B27] Gonzalez-Pena D. Gao G. Baranski M. Moen T. Cleveland B. M. Kenney P. B. (2016). Genome-wide association study for identifying loci that affect fillet yield, carcass, and body weight traits in rainbow trout (*Oncorhynchus mykiss*). *Front. Genet.* 7:203. 10.3389/fgene.2016.00203 27920797 PMC5118429

[B28] Graffelman J. Jain D. Weir B. (2017). A genome-wide study of Hardy–Weinberg equilibrium with next generation sequence data. *Hum. Genet.* 136 727–741. 10.1007/s00439-017-1786-7 28374190 PMC5429372

[B29] Gruhn J. R. Rubio C. Broman K. W. Hunt P. A. Hassold T. (2013). Cytological studies of human meiosis: sex-specific differences in recombination originate at, or prior to, establishment of double-strand breaks. *PLoS One* 8:e85075. 10.1371/journal.pone.0085075 24376867 PMC3869931

[B30] Gutierrez A. P. Yáñez J. M. Davidson W. S. (2016). Evidence of recent signatures of selection during domestication in an Atlantic salmon population. *Mar. Genomics* 26 41–50. 10.1016/j.margen.2015.12.007 26723557

[B31] Gutierrez A. P. Yáñez J. M. Fukui S. Swift B. Davidson W. S. (2015). Genome-wide association study (GWAS) for growth rate and age at sexual maturation in Atlantic salmon (*Salmo salar*). *PLoS One* 10:e0119730. 10.1371/journal.pone.0119730 25757012 PMC4355585

[B32] Guyon R. Rakotomanga M. Azzouzi N. Coutanceau J. P. Bonillo C. D’Cotta H. (2012). A high-resolution map of the Nile tilapia genome: a resource for studying cichlids and other percomorphs. *BMC Genomics* 13:222. 10.1186/1471-2164-13-222 22672252 PMC3441813

[B33] Hayes B. J. (2011). Technical note: efficient parentage assignment and pedigree reconstruction with dense single nucleotide polymorphism data. *J. Dairy Sci.* 94 2114–2117. 10.3168/jds.2010-3896 21427003

[B34] Hosoya S. Kikuchi K. Nagashima H. Onodera J. Sugimoto K. Satoh K. (2017). Genomic selection in aquaculture. *Bull. Jap. Fish. Res. Educ. Agen.* 45 35–39.

[B35] Houston R. D. (2017). Future directions in breeding for disease resistance in aquaculture species. *Rev. Bras. Zootec.* 46 545–551. 10.1590/s1806-92902017000600010

[B36] Joshi R. Woolliams J. Meuwissen T. Gjøen H. (2018). Maternal, dominance and additive genetic effects in Nile tilapia; influence on growth, fillet yield and body size traits. *Heredity* 120 452–462. 10.1038/s41437-017-0046-x 29335620 PMC5889400

[B37] Karayücel I. Ezaz T. Karayücel S. McAndrew B. J. Penman D. J. (2004). Evidence for two unlinked “sex reversal” loci in the Nile tilapia, *Oreochromis niloticus*, and for linkage of one of these to the red body colour gene. *Aquaculture* 234 51–63. 10.1016/j.aquaculture.2003.12.016

[B38] Kocher T. D. Lee W. J. Sobolewska H. Penman D. McAndrew B. (1998). A genetic linkage map of a cichlid fish, the tilapia (*Oreochromis niloticus*). *Genetics* 148 1225–1232. 9539437 10.1093/genetics/148.3.1225 PMC1460020

[B39] Lee B.-Y. Hulata G. Kocher T. D. (2004). Two unlinked loci controlling the sex of blue tilapia (*Oreochromis aureus*). *Heredity* 92 543–549. 10.1038/sj.hdy.6800453 15100706

[B40] Lee B.-Y. Lee W.-J. Streelman J. T. Carleton K. L. Howe A. E. Hulata G. (2005). A second-generation genetic linkage map of tilapia (*Oreochromis* spp.). *Genetics* 170 237–244. 10.1534/genetics.104.035022 15716505 PMC1449707

[B41] Lee S. Kasif S. Weng Z. Cantor C. R. (2008). Quantitative analysis of single nucleotide polymorphisms within copy number variation. *PLoS One* 3:e3906. 10.1371/journal.pone.0003906 19093001 PMC2600609

[B42] Lenormand T. Dutheil J. (2005). Recombination difference between sexes: a role for haploid selection. *PLoS Biol.* 3:e63. 10.1371/journal.pbio.0030063 15736976 PMC1044830

[B43] Li H. (2013). Aligning sequence reads, clone sequences and assembly contigs with BWA-MEM. *arXiv* [Preprint]. https://arxiv.org/abs/1303.3997

[B44] Li H. Handsaker B. Wysoker A. Fennell T. Ruan J. Homer N. (2009). The sequence alignment/Map format and SAMtools. *Bioinformatics* 25 2078–2079. 10.1093/bioinformatics/btp352 19505943 PMC2723002

[B45] Li M. Sun Y. Zhao J. Shi H. Zeng S. Ye K. (2015). A tandem duplicate of anti-müllerian hormone with a missense SNP on the Y chromosome is essential for male sex determination in Nile tilapia, *Oreochromis niloticus*. *PLoS Genet.* 11:e1005678. 10.1371/journal.pgen.1005678 26588702 PMC4654491

[B46] Li Y. Liu S. Qin Z. Waldbieser G. Wang R. Sun L. (2014). Construction of a high-density, high-resolution genetic map and its integration with BAC-based physical map in channel catfish. *DNA Res.* 22 39–52. 10.1093/dnares/dsu038 25428894 PMC4379976

[B47] Liu F. Sun F. Li J. Xia J. H. Lin G. Tu R. J. (2013). A microsatellite-based linkage map of salt tolerant tilapia (*Oreochromis mossambicus* x *Oreochromis* spp.) and mapping of sex-determining loci. *BMC Genomics* 14:58. 10.1186/1471-2164-14-58 23356773 PMC3565888

[B48] Liu S. Vallejo R. L. Palti Y. Gao G. Marancik D. P. Hernandez A. G. (2015). Identification of single nucleotide polymorphism markers associated with bacterial cold water disease resistance and spleen size in rainbow trout. *Front. Genet.* 6:298. 10.3389/fgene.2015.00298 26442114 PMC4585308

[B49] Mair G. C. Scott A. G. Penman D. J. Beardmore J. A. Skibinski D. O. F. (1991). Sex determination in the genus *Oreochromis*: 1. Sex reversal, gynogenesis and triploidy in *O. niloticus*. *Theor. Appl. Genet.* 82 144–152. 10.1007/BF00226205 24213058

[B50] Majumdar K. C. McAndrew B. J. (1986). Relative DNA content of somatic nuclei and chromosomal studies in three genera, *Tilapia, Sarotherodon*, and *Oreochromis* of the tribe Tilapiini (Pisces, Cichlidae). *Genetica* 68 175–188. 10.1007/BF02424441

[B51] Meuwissen T. H. E. Hayes B. J. Goddard M. E. (2001). Prediction of total genetic value using genome-wide dense marker maps. *Genetics* 157 1819–1829. 11290733 10.1093/genetics/157.4.1819 PMC1461589

[B52] NCBI (2018). *Assembly Information by Organism.* Available at: https://www.ncbi.nlm.nih.gov/assembly/organism/8128/all/

[B53] Neira R. (2010). “Breeding in aquaculture species: genetic improvement programs in developing countries,” in *Proceedings of the 9th World Congress on Genetics Applied to Livestock Production*, Leipzig.

[B54] Nielsen H. M. Sonesson A. K. Yazdi H. Meuwissen T. H. E. (2009). Comparison of accuracy of genome-wide and BLUP breeding value estimates in sib based aquaculture breeding schemes. *Aquaculture* 289 259–264. 10.1016/j.aquaculture.2009.01.027

[B55] Ødegård J. Moen T. Santi N. Korsvoll S. A. Kjøglum S. Meuwissen T. H. E. (2014). Genomic prediction in an admixed population of Atlantic salmon (*Salmo salar*). *Front. Genet.* 5:402. 10.3389/fgene.2014.00402 25484890 PMC4240172

[B56] Palaiokostas C. Bekaert M. Khan M. G. Q. Taggart J. B. Gharbi K. McAndrew B. J. (2013). Mapping and validation of the major sex-determining region in Nile tilapia (*Oreochromis niloticus* L.) using RAD sequencing. *PLoS One* 8:e68389. 10.1371/journal.pone.0068389 23874606 PMC3708939

[B57] Palaiokostas C. Kocour M. Prchal M. Houston R. D. (2018). Accuracy of genomic evaluations of juvenile growth rate in common carp (*Cyprinus carpio*) using genotyping by sequencing. *Front. Genet.* 9:82. 10.3389/fgene.2018.00082 29593780 PMC5859378

[B58] Palti Y. Vallejo R. L. Gao G. Liu S. Hernandez A. G. Rexroad C. E. III (2015). Detection and validation of QTL affecting bacterial cold water disease resistance in rainbow trout using restriction-site associated DNA sequencing. *PLoS One* 10:e0138435. 10.1371/journal.pone.0138435 26376182 PMC4574402

[B59] Poletto A. B. Ferreira I. A. Cabral-de-Mello D. C. Nakajima R. T. Mazzuchelli J. Ribeiro H. B. (2010). Chromosome differentiation patterns during cichlid fish evolution. *BMC Genet.* 11:50. 10.1186/1471-2156-11-50 20550671 PMC2896337

[B60] Rastas P. Paulin L. Hanski I. Lehtonen R. Auvinen P. (2013). Lep-MAP: fast and accurate linkage map construction for large SNP datasets. *Bioinformatics* 29 3128–3134. 10.1093/bioinformatics/btt563 24078685 PMC4433499

[B61] Robledo D. Palaiokostas C. Bargelloni L. Martínez P. Houston R. (2017). Applications of genotyping by sequencing in aquaculture breeding and genetics. *Rev. Aquac.* 10 670–682. 10.1111/raq.12193 30220910 PMC6128402

[B62] Roesti M. Moser D. Berner D. (2013). Recombination in the threespine stickleback genome—patterns and consequences. *Mol. Ecol.* 22 3014–3027. 10.1111/mec.12322 23601112

[B63] Seale A. P. Riley L. G. Leedom T. A. Kajimura S. Dores R. M. Hirano T. (2002). Effects of environmental osmolality on release of prolactin, growth hormone and ACTH from the tilapia pituitary. *Gen. Comp. Endocrinol.* 128 91–101. 10.1016/S0016-6480(02)00027-8 12392682

[B64] Shirak A. Seroussi E. Cnaani A. Howe A. E. Domokhovsky R. Zilberman N. (2006). Amh and Dmrta2 genes map to tilapia (*Oreochromis* spp.) linkage group 23 within quantitative trait locus regions for sex determination. *Genetics* 174 1573–1581. 10.1534/genetics.106.059030 16951079 PMC1667067

[B65] Sodeland M. Gaarder M. Moen T. Thomassen M. Kjøglum S. Kent M. (2013). Genome-wide association testing reveals quantitative trait loci for fillet texture and fat content in Atlantic salmon. *Aquaculture* 408 169–174. 10.1016/j.aquaculture.2013.05.029

[B66] Sonesson A. K. Meuwissen T. H. E. (2009). Testing strategies for genomic selection in aquaculture breeding programs. *Genet. Sel. Evol.* 41:37. 10.1186/1297-9686-41-37 19566932 PMC2714299

[B67] Thermo Fisher Scientific Inc (2018). *Axiom^TM^Analysis Suite (AxAS) v4.0 USER GUIDE.* Available at: https://downloads.thermofisher.com/Affymetrix_Softwares/Axiom_Analysis_Suite_AxAS_v4.0_User_Guide.pdf

[B68] Tsai H.-Y. Hamilton A. Tinch A. E. Guy D. R. Bron J. E. Taggart J. B. (2016). Genomic prediction of host resistance to sea lice in farmed Atlantic salmon populations. *Genet. Sel. Evol.* 48:47. 10.1186/s12711-016-0226-9 27357694 PMC4926294

[B69] Tsai H. Y. Hamilton A. Guy D. R. Tinch A. E. Bishop S. C. Houston R. D. (2015a). Verification of SNPs associated with growth traits in two populations of farmed Atlantic salmon. *Int. J. Mol. Sci.* 17:5. 10.3390/ijms17010005 26703584 PMC4730252

[B70] Tsai H.-Y. Hamilton A. Tinch A. E. Guy D. R. Gharbi K. Stear M. J. (2015b). Genome wide association and genomic prediction for growth traits in juvenile farmed Atlantic salmon using a high density SNP array. *BMC Genomics* 16:969. 10.1186/s12864-015-2117-9 26582102 PMC4652364

[B71] Vallejo R. Leeds T. Liu S. Gao G. Welch T. Wiens G. (2015a). “Accuracy of genomic prediction for BCWD resistance in rainbow trout using different genotyping platforms and genomic selection models,” in *Proceedings of the Plant and Animal Genome Conference*, San Diego, CA, 726.

[B72] Vallejo R. Leeds T. Liu S. Gao G. Welch T. Wiens G. (2015b). “Genomic selection for BCWD resistance in Rainbow trout using RADSNP and SNP genotyping platforms, single-step GBLUP and Bayesian variable selection models,” in *Proceedings of the International Symposium on Genetics in Aquaculture*, Townsville, 05796.

[B73] Vallejo R. L. Liu S. Gao G. Fragomeni B. O. Hernandez A. G. Leeds T. D. (2017a). Similar genetic architecture with shared and unique quantitative trait loci for bacterial cold water disease resistance in two rainbow trout breeding populations. *Front. Genet.* 8:156. 10.3389/fgene.2017.00156 29109734 PMC5660510

[B74] Vallejo R. L. Leeds T. D. Gao G. Parsons J. E. Martin K. E. Evenhuis J. P. (2017b). Genomic selection models double the accuracy of predicted breeding values for bacterial cold water disease resistance compared to a traditional pedigree-based model in rainbow trout aquaculture. *Genet. Sel. Evol.* 49:17. 10.1186/s12711-017-0293-6 28148220 PMC5289005

[B75] Varona L. Legarra A. Toro M. A. Vitezica Z. G. (2018). Non-additive effects in genomic selection. *Front. Genet.* 9:78 10.3389/fgene.2018.00078 PMC5845743 29559995

[B76] Vela-Avitúa S. Meuwissen T. H. E. Luan T. Ødegård J. (2015). Accuracy of genomic selection for a sib-evaluated trait using identity-by-state and identity-by-descent relationships. *Genet. Sel. Evol.* 47:9. 10.1186/s12711-014-0084-2 25888184 PMC4339014

[B77] Vilela D. A. R. Silva S. G. B. Peixoto M. T. D. Godinho H. P. França L. R. (2003). Spermatogenesis in teleost: insights from the Nile tilapia (*Oreochromis niloticus*) model. *Fish Physiol. Biochem.* 28 187–190. 10.1023/B:FISH.0000030523.16010.62

[B78] Wang L. Wan Z. Y. Bai B. Huang S. Q. Chua E. Lee M. (2015). Construction of a high-density linkage map and fine mapping of QTL for growth in Asian seabass. *Sci. Rep.* 5:16358. 10.1038/srep16358 26553309 PMC4639833

[B79] Wright P. A. Land M. D. (1998). Urea production and transport in teleost fishes. *Comp. Biochem. Physiol. Part A Mol. Integr. Physiol.* 119 47–54. 10.1016/S1095-6433(97)00407-8 11253818

[B80] Yoshida G. M. Bangera R. Carvalheiro R. Correa K. Figueroa R. Lhorente J. P. (2017a). Genomic prediction accuracy for resistance against *Piscirickettsia salmonis* in farmed rainbow trout. *G3* 8 719–726. 10.1534/g3.117.300499 29255117 PMC5919750

[B81] Yoshida G. M. Lhorente J. P. Carvalheiro R. Yáñez J. M. (2017b). Bayesian genome-wide association analysis for body weight in farmed Atlantic salmon (*Salmo salar* L.). *Anim. Genet.* 48 698–703. 10.1111/age.12621 29044715

[B82] Yoshida G. M. Carvalheiro R. Rodríguez F. H. Lhorente J. P. Yáñez J. M. (2018). Single-step genomic evaluation improves accuracy of breeding value predictions for resistance to infectious pancreatic necrosis virus in rainbow trout. *Genomics* 10.1016/j.ygeno.2018.01.008 [Epub ahead of print]. 29357303

