# Supplementary material for: Development and Validation of 58K SNP-Array and High-Density Linkage Map in Nile Tilapia (O. niloticus)
Source: Front Genet. 2018 Oct 15;9:472. doi: 10.3389/fgene.2018.00472 (PMC6196754; doi:10.3389/fgene.2018.00472)
Supplement: Supplementary file 1 [file Table_1.PDF]

Supplementary Material

Development and validation of 58K SNP-array and high-density linkage map in Nile tilapia (O. niloticus)

Rajesh Joshi[1,*], Mariann Árnyasi[1], Sigbjørn Lien[1], Hans Magnus Gjøen[1], Alejandro Tola Alvarez[2], Matthew Peter Kent[1]

[1]Department of Animal and Aquacultural Sciences, Faculty of Biosciences, Norwegian University of Life Sciences, N-1432, Ås, Norway

[2]Genomar Genetics AS, Norway

*** Correspondence:** Rajesh Joshi: rajesh.joshi@nmbu.no

Supplementary Table 1: Observations in each factorial mating in Group 1 population. 11 different sires (M1 to M11) are mated with 8 different dams (F1 to F8) in factorial manner. Only full-sib families ≥ 8 offspring are shown.

	**F1**	**F2**	**F3**	**F4**	**F5**	**F6**	**F7**	**F8**	**Total**
**M1**	22	13	10	-	8	-	-	-	**53**
**M2**	-	-	-	10	-	21	10	24	**65**
**M3**	-	-	-	12	-	23	11	16	**62**
**M4**	-	-	-	13	-	9	-	-	**22**
**M5**	11	24	9	-	8	-	-	-	**52**
**M6**	-	-	-	-	-	18	8	13	**39**
**M7**	19	12	-	-	-	-	-	-	**31**
**M8**	-	-	-	14	-	9	12	28	**63**
**M9**	-	14	-	-	10	-	-	-	**24**
**M10**	16	14	8	-	8	-	-	-	**46**
**M11**	-	-	-	11	-	-	-	-	**11**
**Total**	**68**	**77**	**27**	**60**	**34**	**80**	**41**	**81**	**468**

Supplementary Table 2: Observations in different full-sib families in the Group 2 population.

**Dam**	F9	F10	F11	F12	F13	F14	F15	**Total**
**Sire**	M12	M13	M14	M15	M16	M17	M18	
**No. of offspring**	22	24	26	36	37	37	39	**221**

Supplementary Table 3: Published linkage maps for Tilapia species

Species of Tilapia		Map length (cM)	Marker number and type	Average marker interval (cM)	Authors & Year
Oreochromis niloticus		704	62 microsatellites + 112 AFLP	-	(Kocher et al., 1998)
O. niloticus X *O. aureus*		1,311	525 microsatellite and 21 gene-based markers	2.4	(Lee et al., 2005)
O. niloticus		34,084 cR_{3500} and 937,310 kb	1358 markers – radiation hybrid (RH) map	742 Kb	(Guyon et al., 2012)
O. niloticus		1,176	3,802 SNPs	0.7	(Palaiokostas et al., 2013)
O. mossambicus	Female	514	13 microsatellites and 49 AFLPs	8.3	(Agresti *et al.*, 2000)
	Male	1632	60 microsatellites and 154 AFLPs	7.6	
O. mossambicus		1042.5	301 markers		(Liu *et al.*, 2013)
O. mossambicus X *O. spp.* (Saline tilapia)	Consensus	1067.6	401 microsatellites including 282 EST-derived markers	3.3	
	Male	950.8	261 markers	3.6	
	Female	1030.6	261 markers	4	
Red tilapia		984.0	320 markers	3.1	

Supplementary Table 4: Summary statistics showing the inter-marker distance (base pairs) of the SNPs on the Onil50-array.

Orenil1.1 assembly			O_niloticus_UMD1 assembly		
**LG**	**Mean**	**sd**	**LG**	**Mean**	**sd**
**LG01**	12124	4821	**LG01**	13548	10950
**LG02**	12253	6180	**LG02**	14666	14189
**LG03**	13646	10919	**LG03a**	20689	35557
			**LG03b**	38045	55867
**LG04**	12522	7061	**LG04**	15509	16232
**LG05**	12771	6860	**LG05**	13564	8909
**LG06**	12701	6249	**LG06**	15149	15972
**LG07**	12356	5419	**LG07**	13227	9624
**LG08-24**	12702	7093	**LG08**	13326	10639
**LG09**	12091	5667	**LG09**	14377	16435
**LG10**	12068	4742	**LG10**	17227	16536
**LG11**	12608	6704	**LG11**	13642	11567
**LG12**	12595	6466	**LG12**	14516	14585
**LG13**	12377	5895	**LG13**	14188	14768
**LG14**	12652	6626	**LG14**	14634	13260
**LG15**	12239	6557	**LG15**	15991	13433
**LG16-21**	12556	6226	**LG16**	15323	17131
**LG17**	12154	5881	**LG17**	14153	12842
**LG18**	12623	7246	**LG18**	15992	22063
**LG19**	12196	5849	**LG19**	13514	12128
**LG20**	12632	6502	**LG20**	13910	11003
**LG22**	12603	7125	**LG22**	16212	18656
**LG23**	12934	8374	**LG23**	19813	22055
**Across genome**	12505	6538	**Across genome**	15410	17712
**Scaffolds**	36493	22508	**Scaffolds**	28097	31755
**mito**	1636	1057	**mito**	1636	1057

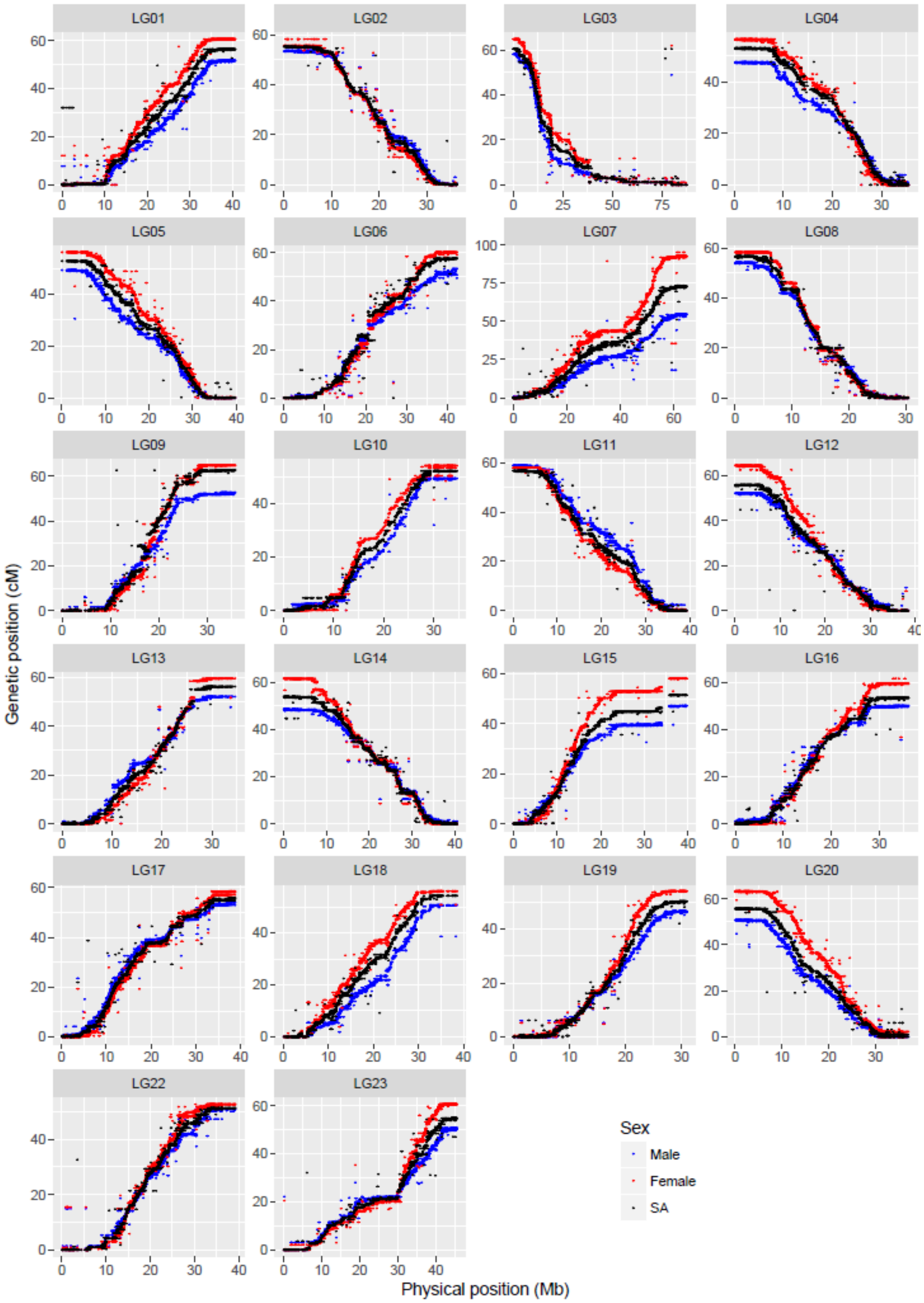

Supplementary Figure 1: Comparison of map positions between genetic and physical maps for different LGs in Build1 linkage map. The y-axis gives the linkage map positions, and the x-axis gives the physical positions. Linkage groups and the physical positions are based on O_niloticus_UMD1 Assembly. The maps are color-coded: red for female specific, blue for male specific and black for sex-averaged linkage maps. Inversion in maps shows that the genetic order is inverted.

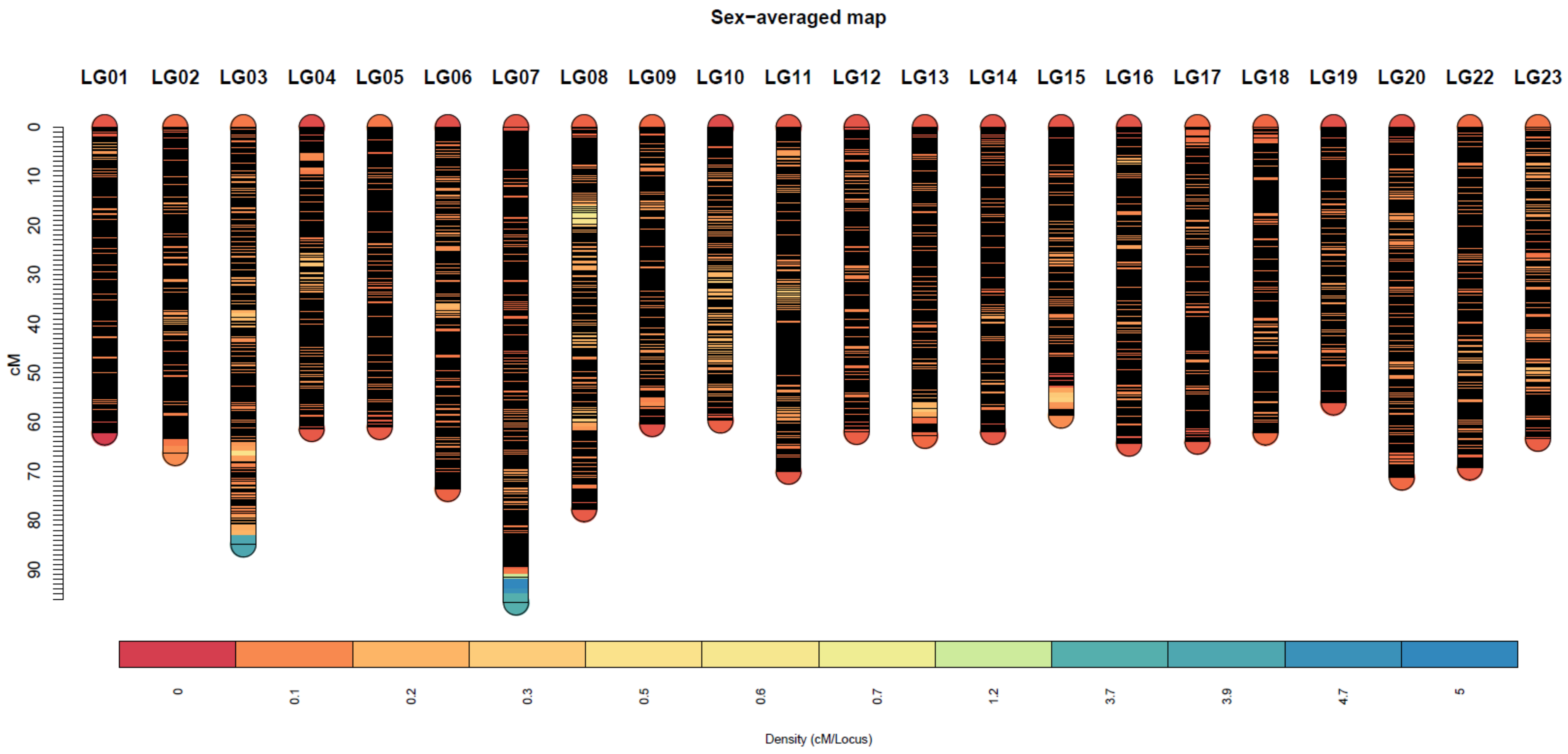

Supplementary Figure 2: The high-density consensus (sex-averaged) Build2 linkage map for Nile tilapia. The density is measured as cM/locus (higher the value, lower the number of markers in that locus)

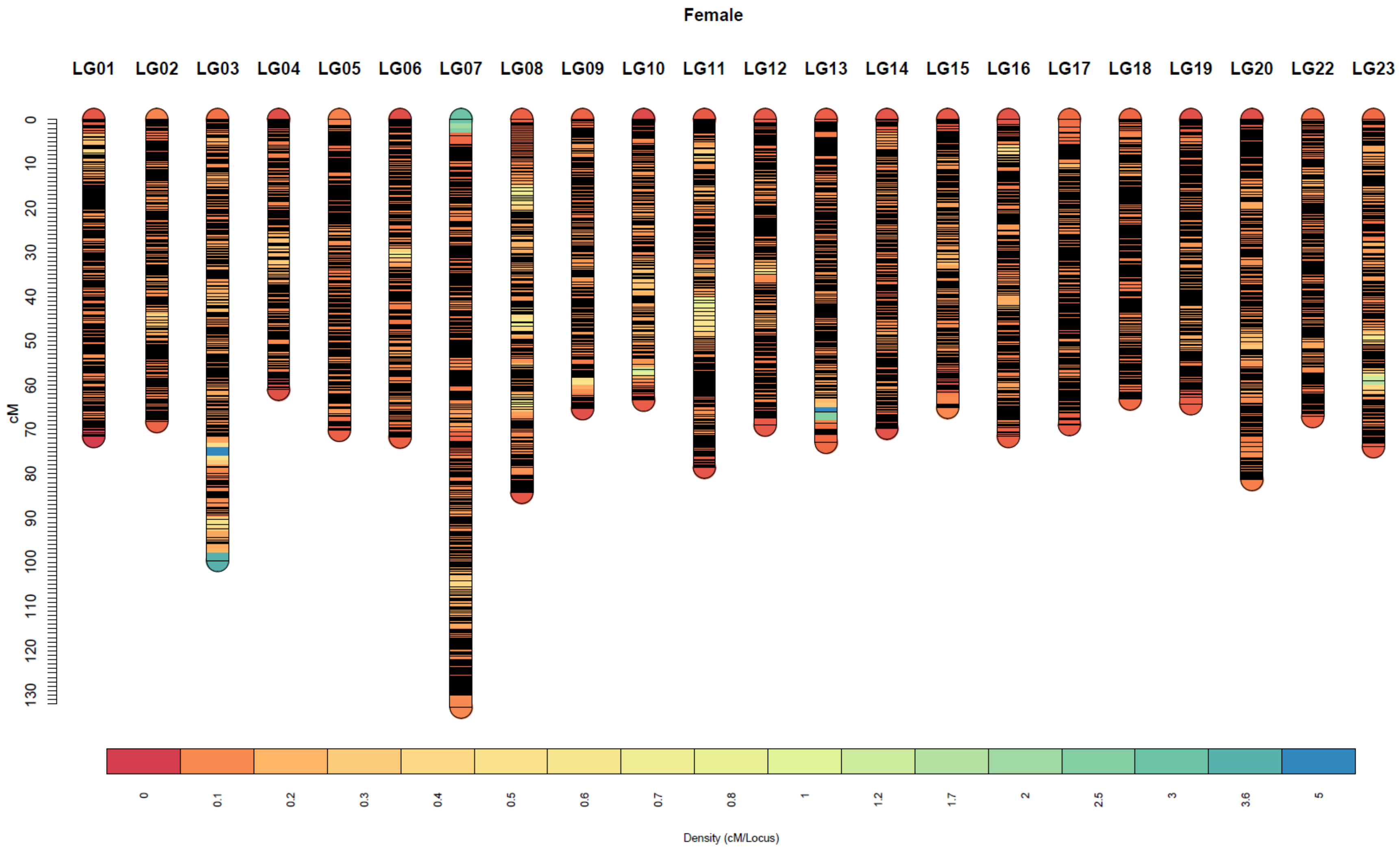

Supplementary Figure 3: The high-density female sex specific Build2 linkage map of Nile tilapia. The density is measured as cM/locus (higher the value, lower the number of markers in that locus)

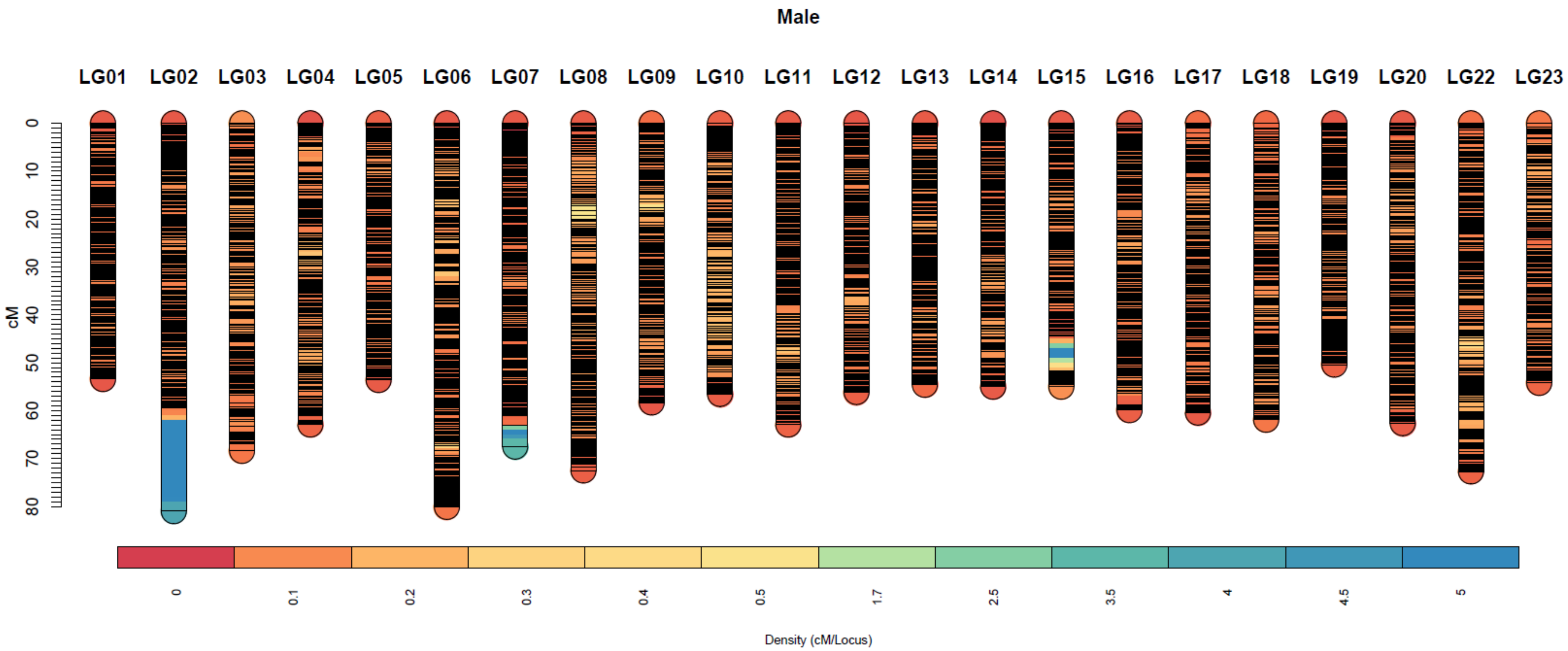

Supplementary Figure 4: The high-density male sex specific Build2 linkage map of Nile tilapia. The density is measured as cM/locus (higher the value, lower the number of markers in that locus

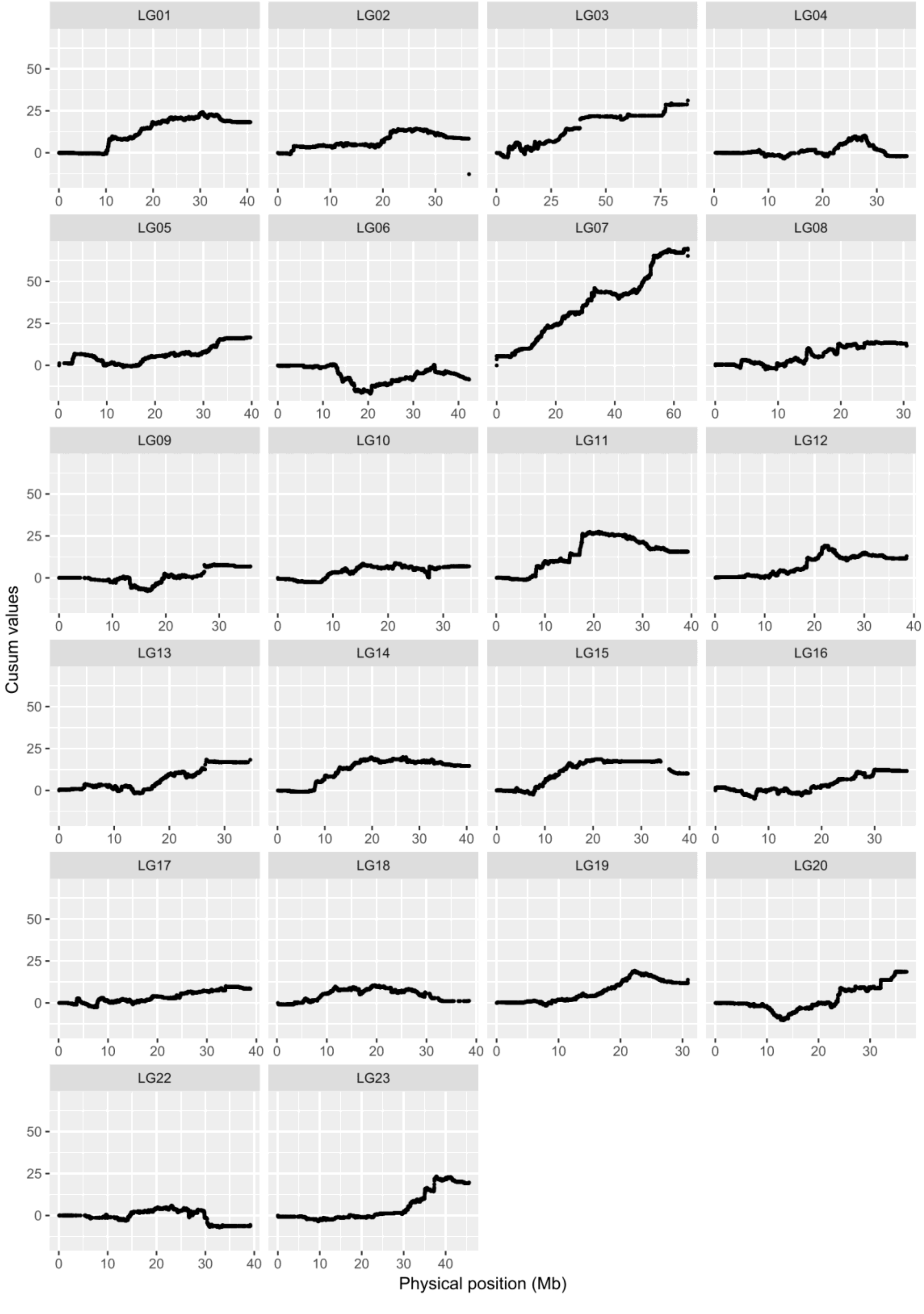

Supplementary Figure 5: Cusum plots indicating the sex- patterned differentiations in the genome of tilapia. Cusum is a time series technique to emphasise shifts. Flat lines represent no difference between female and male recombination rate. Upward vertical lines represent more recombination rate in female LGs and downward vertical lines represent more recombination rate in male LGs.

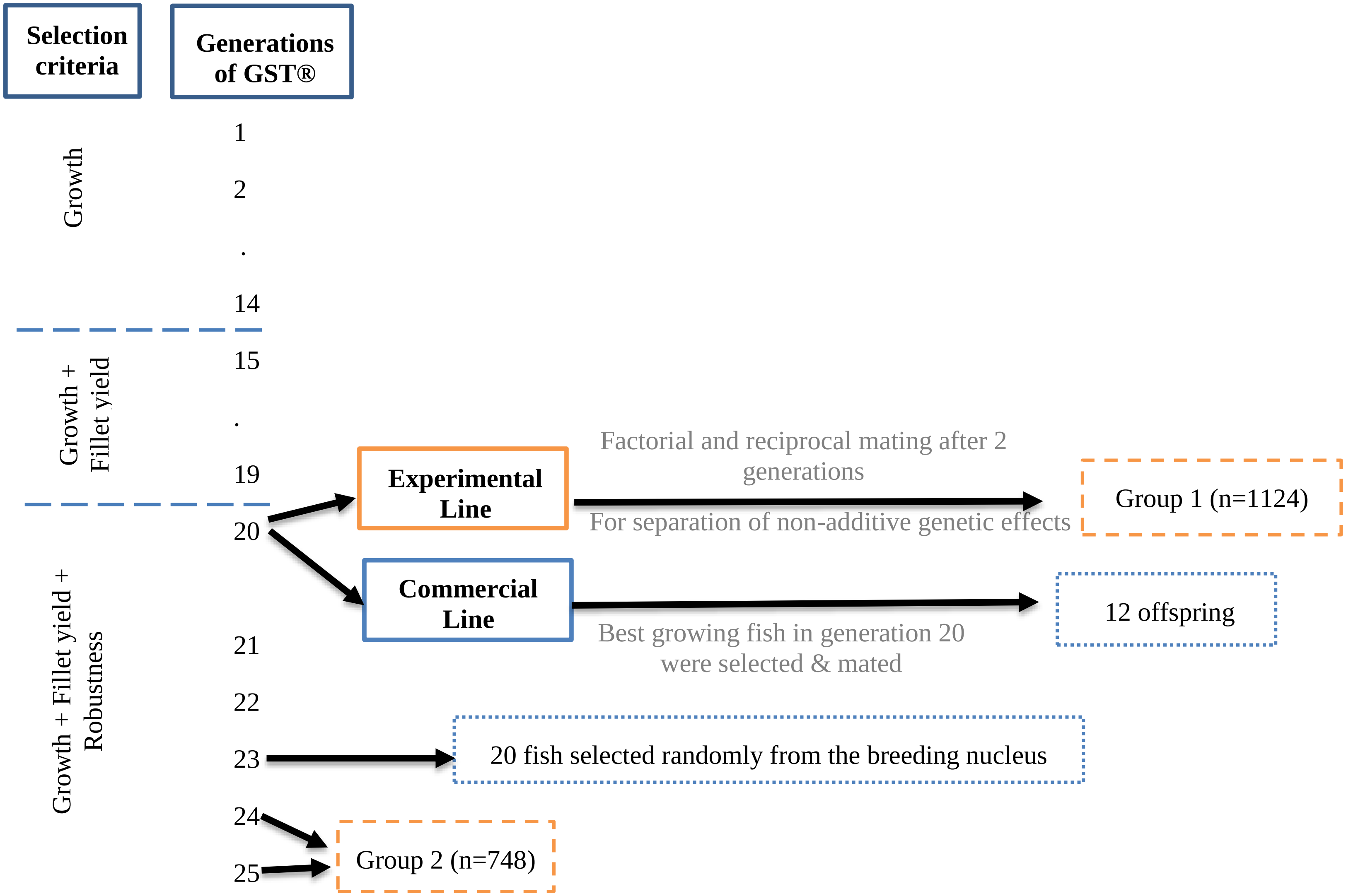

Supplementary Figure 6: Selection criteria, sampling and genotyping design. Fish from Group 1 and Group 2 were used for linkage map construction. Group 1 (n=1124) comprised individuals collected following the branching of the 20th generation, and were factorially crossed against each other after 2 generations. Fish from Group 2 (n=748) were obtained from the 24th and 25th generations of GST®. 12 fish from commercial line and 20 fish from generation 23 were used for whole genome sequencing. Fish from group 1, Group 2 and 3119 fish from generations 20, 21 and 25 of breeding nucleus were used for array performance and validation.
